# Effects of pain neuroscience education combined with neuromuscular exercises on pain, functional disability and psychological factors in chronic low back pain: A study protocol for a single-blind randomized controlled trial

**DOI:** 10.1371/journal.pone.0309679

**Published:** 2024-11-04

**Authors:** Ehsan Alvani, Bahram Sheikhi, Amir Letafatkar, Giacomo Rossettini

**Affiliations:** 1 Faculty of Physical Education and Sport Sciences, Department of Biomechanics and Sport Injuries, Kharazmi University, Tehran, Iran; 2 School of Physiotherapy, University of Verona, Verona, Italy; 3 Faculty of Sport Sciences, Department of Physiotherapy, Universidad Europea de Madrid, Calle Tajo s/n, Villaviciosa de Odón, Spain; Monash University, AUSTRALIA

## Abstract

**Objective:**

Chronic low back pain (CLBP) is a prevalent health condition worldwide. Several therapeutic interventions aim to improve CLBP. Pain Neuroscience Education (PNE) helps patients better understand their pain from biological and physiological perspectives, which clinicians use to reduce pain and disability in patients with chronic musculoskeletal conditions. Neuromuscular exercises (NMS) are also treatments adopted in CLBP. This study will investigate whether PNE combined with an NMS program improves pain, functional and psychological outcomes more than NMS alone in patients with CLBP.

**Methods:**

In this single-blind randomized controlled trial, 60 patients (male and female; age range, 30–60 years) diagnosed with CLBP will be randomly assigned to one of the following groups: (1) PNE plus NMS (n = 30; 24 sessions of PNE plus NMS in a total of 8 weeks, 3 each week), and (2) NMS alone (n = 30; 24 sessions of NMS sessions in a total of 8 weeks, 3 each week). Outcome assessors will be blinded to the group allocation. The primary outcome will be pain. Secondary outcomes will be disability, fear-avoidance beliefs about work and physical activity, self-efficacy, exercise anxiety, and kinesiophobia. Outcomes will be assessed at baseline, after 8 weeks of intervention, and 6 months post-intervention.

**Discussion:**

The findings of this RCT will help shed light on new treatment strategies to address the biopsychosocial dimensions of CLBP. The study protocol will be conducted in a clinical setting, offering the opportunity for future implementation in healthcare systems. Moreover, it will help clarify whether a combined treatment (PNE with NMS) is more effective than NMS alone for improving pain, functional and psychological outcomes in CLBP.

**Trial registration:**

**Study registration**: The study was prospectively registered in the Iranian Registry of Clinical Trials—IRCT20190427043384N2 (https://www.irct.ir/trial/69146). Registered on March 17, 2023.

## Introduction

Chronic low back pain (CLBP) is a multifactorial disease that burdens the global healthcare system [1,2], resulting in pain, disability [3], stiffness, and fear of movement [4]. Approximately 80%-90% of people worldwide experience some form of LBP [4,5], making it one of the most common reasons for seeking healthcare in low- and middle-income countries [6]. Along with neck pain, CLBP is a medical condition associated with the highest overall costs [3] that impact the biological, psychological, and social dimensions of life [7].

Ssystematic reviews [8] and Cochrane reviews [9] recommend nonsurgical treatments for CLBP, including exercise therapy and education [10]. However, chronic pain is a complex phenomenon leading to changes in the central nervous system (CNS) that challenge the efficacy of CLBP treatment, offering the opportunity to analyze newer therapeutic approaches [11–13]. Recent evidence in patients with chronic musculoskeletal pain has revealed that brain plasticity induces central sensitization (hyperexcitability of the CNS), which alters pain processing and creates pain memory and kinesiophobia [1,14,15]. These CNS changes can exacerbate anxiety, depression, stress, and pain catastrophizing [16], leading to a vicious cycle of pain, psychological issues, activity avoidance, reduced functionality, weight gain, and persistent pain [14].

Pain neuroscience education (PNE) [17,18] aims to change patients’ conceptualization of pain, educating them about the neurobiology and neurophysiology of pain and focusing on the peculiarity and singular variances in the overall pain experience [14–17]. Recent systematic reviews and meta-analyses have reported that PNE helps reduce pain, improve pain knowledge, enhance function, and lower disability and psychosocial distress [19–21]. Moreover, PNE increases pain thresholds during physical activity and exercise and minimizes healthcare utilization [19–21]. Studies have investigated the effect of PNE combined with various treatments (e.g., therapeutic exercise) with positive results [19]. For example, PNE combined with motor control training was more effective than core stability training in improving disability and pain [22]. These findings indicate that PNE has clinical value but also suggest the importance of continuing its study in combination with other types of exercise [14,16].

In CLBP, various types of therapeutic exercises are suggested as treatments (e.g., strengthening, stretching, core stability, McKenzie, yoga, and functional restoration) [23,24]. According to a Cochrane review [25], the effect of these exercises on CLBP is supported by moderate certainty of evidence. Neuromuscular Exercises (NMS) represent an under-investigated area of CLBP [26]. The general aim of NMS is to restore pain-induced impairments and increase functional activities to improve coordination, power, range of motion, and proprioception in patients with CLBP [27]. Although previous RCTs have reported positive effects of NMS on CLBP, showing improvements in lumbar muscle control, flexibility, and strength [27–29], the generalizability of their findings is limited because they mainly focused on healthcare providers (e.g., nurses) as patients. Moreover, a recent review emphasized that the methodological quality of RCTs on NMS is poor (e.g., in terms of outcome reporting, blinding of the assessor, and intention-to-treat analysis), and further investigation is needed [26].

Although PNE [30] and NMS [27–29] have been previously investigated alone, RCTs on their combined effects on CLBP are lacking. Existing RCTs on NMS have mainly investigated the effect of exercise on pain and disability outcomes rather than psychological ones [26]. This gap provides an opportunity for new research that combines PNE with NMS to address both the physical and psychological impairments in CLBP. Recent evidence corroborates our methodological choice, suggesting that the addition of PNE to other treatments, such as exercise, leads to greater clinical improvements than a multimodal approach alone, particularly in influencing psychosocial variables [31,32]. Accordingly, this study aims to evaluate the efficacy of a combined PNE and NMS program compared to NMS alone in improving pain, functional and psychological outcomes in patients with CLBP. We hypothesize that PNE plus NMS will improve pain, disability, and psychological outcomes in patients with CLBP when compared to NMS alone.

## Methods

### Study design

**Fig 1:**
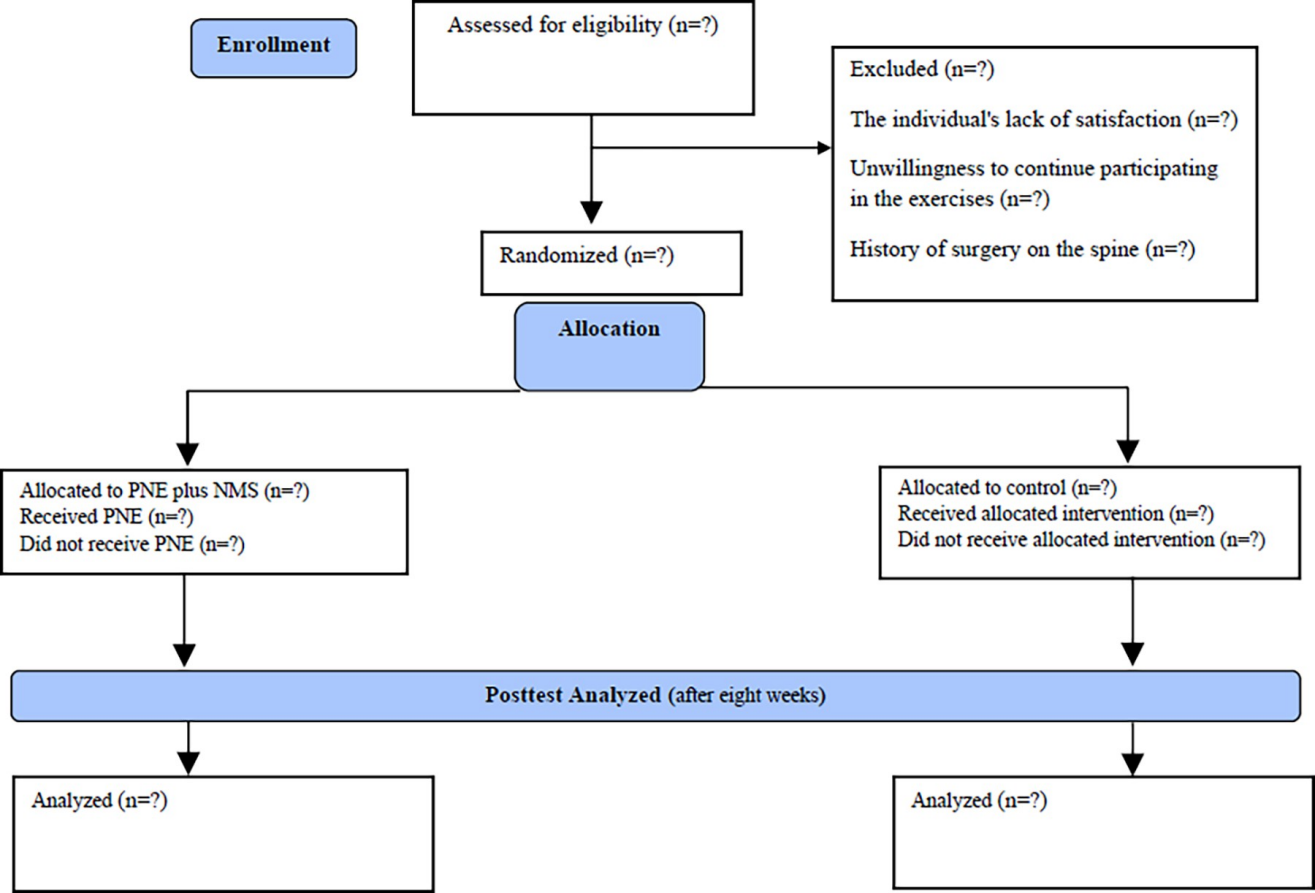
CONSORT diagram.

This study will be a single-blind assessor randomized clinical trial (RCT) with two arms (group 1: Experimental-PNE plus NMS; group 2: Control-NMS) comparing the outcomes between intervention groups in patients with CLBP. The Standard Protocol Items: Recommendations for Interventional Trials (SPIRIT) reporting guidelines are used to report this protocol [33,34] and are presented in S1 File. This study will follow the Consolidated Standards of Reporting Trials guidelines (CONSORT) for RCT [35]. The interventions will be presented following the Template for Intervention Description and Replication (TIDieR) checklist [36] reported in S2 File. A study flowchart is presented in Table 1 and Fig 1 [37].

**Table 1:** Recommended protocol items scheduled for enrolment, interventions, and assessments according to evidence [37].

	ENROLMENT	ALLOCATION	BASELINE	AFTER 8 WEEKS (PNE) PLUS (NMS)				
TIMEPOINT	-t_1_	0	Pr_1_	Po_1_				
**ENROLMENT**:								
Eligibility screen	X							
Informed consent	X							
[Randomization]		X						
Allocation		X						
**INTERVENTIONS**:					X			
					X			
					X			
[PNE+ NMS]					X			
[BS]					X			
**ASSESSMENTS:**								
[Research Diagnostic Criteria CLBP]	X							
[VAS]	X		X		X			
[ODI]			X		X			
[FABQ]			X		X			
[PSEQ]			X		X			
[TSK]			X		X			

**Abbreviation**: PNE: Pain Neuroscience Education, NME: Neuromuscular Exercise, CLBP: Chronic low back pain, NMS: Neuromuscular exercises, Po_1-2-3_: Post-test, Pr_1_: Pre-test, Visual Analog Scale (VAS), ODI: Oswestry Disability Index, FABQ: Fear-Avoidance Beliefs Questionnaire, Pain Self-Efficacy Questionnaire (PSEQ), Tampa Scale for Kinesiophobia (TSK).

### Ethical aspects

Ethical approval has been obtained from the Ethics Committee on Sports Sciences Research Institute of Iran (Approval ID: IR.SSRC.REC.1401.081). The protocol has been prospectively registered at the Iranian Registry of Clinical Trials (IRCT20190427043384N2; **https://www.irct.ir/trial/69146**).

### Setting and participants

Before starting the project, all patients will complete and sign an informed consent form based on the latest revision of the Declaration of Helsinki [38]. The population considered for this research will comprise Persian patients aged 30–60 years with CLBP. Participants will be informed about this study and how to register through advertisements in physical therapy centres, research group networks, and social media. A physiotherapist and a sports injury specialist will recruit and register patients in a physiotherapy clinic. Then, on a certain date, the registration of all patients in the health centre and human performance laboratory of Kharazmi University will be completed in person, and qualified people will be included in the study. Among the participants, 60 will be randomly divided into Group 1, Experimental-PNE plus NMS (n = 30) and Group 2 Control-NMS (n = 30). Participants will be informed that they are free to withdraw consent at any time during the study.

#### Inclusion criteria

We will include participants who satisfy the following criteria: (a) Persian-native speaker (male and female); (b) presenting CLBP diagnosed by an experienced physiotherapist; (c) with complaints of more than 3 months; (d) with a pain intensity of LBP ≥ 5 on the Visual Analog Scale (VAS) [39]; and (e) with a pattern reported between the lower ribs and the creased part of the buttocks without nonspecific pathoanatomical cause [32].

#### Exclusion criteria

We will exclude participants with: (a) a current substance use disorder, such as smoking or alcohol, or a history of hospitalization for treatment of a substance use disorder: (b) any significant comorbidities or issues that may interfere with the study or have deleterious effects on the participant, as determined by the investigators; (c) a history of spine fracture and surgery; and (d) any specific pathology in the spine such as disc herniation, spina bifida and spondylolisthesis based on radiographic examination and assessed by a physician [23,32,40,41]. Additionally, participants with a history of recurrent LBP [42], or those who habitually practice exercise for more than 30 minutes per week in the 6 months before the study will be excluded [43].

### Procedure

All demographic information and inclusion and exclusion criteria will be collected through relevant questionnaires. A physiotherapist specializing in the musculoskeletal field with 5 years of clinical practice will examine all patients to ensure that the selection process follows the inclusion and exclusion criteria. If the patient is interested in participating in the study, an assessor blinded to the group allocation will collect baseline data after signing the research-controlled informed consent. After the initial assessment, patients will be randomly assigned to one of two study groups (Group 1 Experimental-PNE plus NMS; Group 2 Control-NMS).

### Randomization

The principal investigator (E.A.) will generate the allocation sequence using an online random number generator (Random.org). Patients will be randomly assigned to one of two groups in a ratio of 1:1 as follows: Group 1 Experimental-PNE plus NMS (n = 30) or Group 2 Control–NMS (n = 30). Randomization will draw numbers from 1 to 60, prepared in advance and placed in sealed opaque envelopes in a box (Fig 1). Participants will be told which intervention they were randomized to at the end of the study [44–46].

### Interventions

The groups will receive different treatment programs as follows:

- In Group 1 Experimental-PNE plus NMS, the intervention will be offered three days a week for up to 8 weeks;
- In Group 2 Control–NMS, the intervention will be administered three days a week for up to 8 weeks.

The interventions will be delivered face-to-face in an exercise room of a physical therapy clinic. Each session will be provided individually by a physical therapist with an average of 5 years of experience in musculoskeletal pain.

### Intervention description

#### PNE training

In this study, PNE will be adopted to reduce the threat value of pain, increase participants’ knowledge of pain, and reframe their perception of pain [47]. Participants should understand that all pain is generated, synthesized, and controlled by the brain and that pain symptoms are often associated with CNS hypersensitivity without tissue damage [14]. Moreover, PNE aims to reconceptualize patients’ negative beliefs about pain [47], which may arise from unhelpful diagnostic, prognostic, or therapeutic conclusions. PNE sessions will also focus on providing information about the nature of pain to reduce kinesiophobia, fear-avoidance beliefs, and non-adaptive behaviors, thereby promoting self-efficacy [14,48].

PNE will cover 8 topics: (a) neurophysiology of pain, (b) nociception, (c) nociceptive pathways, (d) neurons and synapses, (e) action potential, (f) spinal inhibition and facilitation, (g) sensitization, and (h) plasticity of the nervous system [30]. Participants will receive a booklet with detailed written explanations and illustrations of pain physiology and CNS sensitization processes. The training content will be adapted from the book "Explaining Pain", which cover the physiology of the CNS with a focus on the pain system [14,43,47]. PNE will be delivered both verbally (through oral explanations by the physiotherapist) and visually (using summaries, pictures, and diagrams on a computer and paper) [7,14,47]. Combining different educational strategies will guarantee a more effective learning experience by adopting multiple channels simultaneously [49].

Patients with CLBP will participate in face-to-face PNE sessions provided by a physiotherapist specializing in the musculoskeletal field who has received 20 hours of training on PNE and has 5 years of clinical experience. PNE sessions will be offered 3 times a week for 10–20 minutes [50] before the NMS sessions [30]. Each week, a specific topic will be discussed in individual sessions (Table 2). Each session will begin with a brief recap of the previously discussed topic, followed by new topic. If a patient misses one or more sessions, they will have the opportunity to catch up, with the schedule adjusted flexibly to ensure continuity.

**Table 2:** Details of the pain neuroscience education according to evidence [30].

Week	Topic of PNE	Description of contents session	Aims	Details of class
1	Neurophysiology of pain	Session 1: • What is pain? • How pain is created? (CNS, nerve pathways, acute and chronic pain/ injuries) Session 2: • CNS function in chronic pain (Central sensitization, Allodynia, Hyperalgesia) • Neurobiology and neurophysiology of pain and pain processing Session 3: • How do you treat your pain? • How can the pain be managed?	• Improving patient’s knowledge of pain mechanisms • Decrease fear related to movement • Teach patients to rethink the way they view pain • Increase pain thresholds during exercise and all activities	• 10 to 20 min • face-to-face education
**2**	Nociception	Session 1: • What is pain? • Explain and compare concepts and differences between acute and chronic pain with real-world Session 2: • How do you treat your pain? • How can the pain be managed? Session 3: • Emphasize the importance of exercise and decrease fear related to movement • Teach patients to rethink the way they view pain and increase pain thresholds during exercise and all activities	• Improving patient’s knowledge of pain mechanisms • Decrease fear related to movement • Teach patients to emphasize the importance of exercise • Increase pain thresholds during exercise and all activities	• 10 to 20 min • face-to-face education
**3**	Nociceptive pathways	Session 1: • How pain is created? (CNS, nerve pathways, acute and chronic pain/ injuries) • CNS function in chronic pain (Central sensitization, Allodynia, Hyperalgesia) Session 2: • Explain the paths of pain sensation and noxious pain sensations • Highlights how psychological aspects and thoughts affect pain Session 3: • Re-education of patient misconceptions regarding CLBP (wear and tear) • Talking and discussing the patients’ pain story • Asking questions like: “Why do you think exercise is dangerous for you?” or “What do you think will happen when you perform the exercise?”	• Follow previous aims • Facilitating movement and decreasing healthcare consumption • Increased patient’s Self-esteem about pain self-management • Encouraging the patients to exercise to heal pain	• 10 to 20 min • face-to-face education
**4**	Neurons and synapses	Session 1: • Repeat previous contents Session 2: • Explain the structure and function of neurons and synapses Session 3: • The processes of electrical signals, and how the patient’s thoughts can influence these signaling processes	• Follow previous aims • Facilitating movement and decreasing healthcare consumption • Increased patient’s Self-esteem about pain self-management • Encouraging the patients to exercise to heal pain • Improving patient’s thoughts	• 10 to 20 min • face-to-face education
**5**	Action potential	Session 1: • Repeat previous contents Session 2: • Describe action potentials Session 3: • Describe thresholds, and differences in sensations felt by each person by comparing them to signals and alarms generated by the body	• Repeat previous contents • Ensure achieved the goals • Improving patient’s knowledge of action potentials • Teach patients differences in sensations	• 10 to 20 min • face-to-face education
**6**	Spinal inhibition and facilitation	Session 1: • Repeat previous contents Session 2: • Explain the process of inhibition and promotion of the spine by comparing the process of electrical signal transmission Session 3: • Emphasize the change in sensation according to the state of the body	• Repeat previous contents • Facilitating process of inhibition • Emphasize the change in sensation according to the state of the body	• 10 to 20 min • face-to-face education
**7**	Sensitization	Session 1: • Repeat previous contents Session 2: • Explain the types of sensations • Explain the process of transmitting each sensation, and the differences Session 3: • Talking and discussing about the control theory	• Follow previous aims • Improving patient’s knowledge of pain mechanisms • Improving patient’s knowledge of the types of sensations • Discussing about the control theory	• 10 to 20 min • face-to-face education
**8**	Plasticity of the nervous system	Session 1: • Repeat previous contents Session 2: • What is neuroplasticity? • Explain the basic concept of neuroplasticity in relation to changes in the brain caused by experience and learning Session 3: • Guidance about lifestyle and movement modifications • Talking and discussing the patients’ pain story • Asking questions like: “What are the positive changes in exercise for you?” or “How do you think you feel when you exercise?”	• Follow previous aims • Improving patient’s knowledge of pain mechanisms • Teach patients to rethink the way they view pain • Increase pain thresholds during exercise and all activities • Personalization of the patient’s pain story • Ensuring change in the patient’s perspective of her/ his pain story • Encourage the active lifestyle of the patient • Ensure achieved the goals	• 10 to 20 min • face-to-face education

The Neurophysiology of Pain Test [47] will be used to examine whether the patient understands PNE. Patients will complete the Neurophysiology of Pain Test before and after PNE sessions [47], with their level of understanding determined by their scores. Research has shown that patients with a higher level of education are more likely to improve their pain biology knowledge after PNE sessions [51,52]. Therefore, we will consider the influence of this variable. Based on the study of Thomas Bilterys et al.[52], participants will be grouped by their level of education and treatment arm to compare the treatment’s effectiveness in individuals with higher versus lower education. Educational attainment will be used to divide the participants in both the experimental and control group into subgroups based on their self-reported level of education: "What is the highest education degree you have received?". Participants with at least a Bachelor’s degree will be allocated to the higher education group, while others will be allocated to the lower education group [52].

#### NMS training protocol

The NMS protocol will follow evidence in the CLBP field [23,27–29,53,54]. Each training session will consist of three stages.

- Warm-up phase: this phase will last for 10 minutes and include stretching exercises and gentle running in the gym.
- Central phase: this phase will last for 45 minutes and includes specific exercises aimed at ameliorating spine stability, improving trunk muscle endurance, balance and posture control. Additionally, the exercise will focus on enhancing the strength of back muscles, and increasing the range of motion of the back and pelvis according to the Suni et al. protocol (Table 3) [28,29,54,55].
- Cool-down phase: in this phase, patients will perform gentle exercises and stretching for 5 minutes.

**Table 3:** Neuromuscular exercise protocol according with evidence [23,27–2,9,53,54].

Aims	EXERCISE TYPE	REPETITIONS	LEVEL 1 (WEEK 1–3)	LEVEL 2 (WEEK 4–6)	LEVEL 3 (WEEK 7–8)
**1. Increase spinal stability using exercises which minimize the load on spinal**	**Modified curl-up (“McGill curl-up”)** Supine lying, one knee in flexion, the other leg stretched out, and hands under lumbar lordosis. Low curl-ups without pressing the lumbar spine down.	6–8 repetitions to each side	Elbows on the floor	Elbows off the floor	Elbow towards opposite, flexed hip joint
	**Bird dog** Four-point kneeling, pressing the hands and shins towards the floor (stable shoulder region, neutral lumbar spine).	6–8 repetitions to each side	Stretching one leg out on the floor	Lifting the leg to same level with the trunk	The opposite arm is placed simultaneously with the leg. Progression: hold up for two breaths
	**Side bridge or Mermaid** Side-lying with bent knees, pressing supporting forearm on the floor, or side-sitting with upper knee upward, pressing supporting hand down (shoulder stability).	6–8 repetitions to each side	Lifting the pelvis up	Longer lever arm by stretching the lower leg	Longer lever arm with straight legs
**2. Improve endurance of the trunk musculature**	**Single leg stretch**	8–10 repetitions to each side	Supine crook lying. Stretching one leg on the floor and drawing it back while maintaining a neutral spine.	Head and shoulders off the mat, drawing one leg to the chest and stretching the other leg on the floor. Switching the position of the legs and maintaining a neutral spine.	As in Level 2, the extended leg is stretched above the floor.
	**Shoulder bridge**	6–8 repetitions	Supine crook lying. Lifting up of the pelvis while maintaining a neutral spine.	Maintaining the bridge position and neutral spine, lifting one heel of the floor, without pelvic rotation. • 5 repetitions with both legs	As in Level 2, the leg is lifted up to a 90°angle in the hip and knee joints. • 5 repetitions with both legs
**3. Improve balance, postural control and co-contraction of the stabilizing muscles around lumbar NZ in various up-right postures and movements**	**Weight transfer, side lunge and one leg stand**	8–10 repetitions	Weight transfer from side to side, lining in the legs, maintaining neutral lumbar spine and light rotation in the thoracic spine.	Side lunge maintaining neutral spine. Weight shift on the other side. • 6–8 repetitions	From the side lunge, take off the bending leg to a one-leg stand. Progression: Taking off the leg from the floor first from a narrow position and minor side lunge to a wider position and deeper side lunge. • 6–8 repetitions to each side
	**“Tai chi warrior”** One-leg squat stretching the other leg back → lifting the same leg to the front of the body with the knee flexed. Maintain a neutral spine throughout the movement, and control the hip in the supporting leg.	6 repetitions to each leg	The left leg is left forward while bending the left knee to 90° while straightening the right leg to the rear and keeping the hands on the hips; 2) standing straight on the left leg while raising the right foot and bending the right knee 90°; and 3) repeating on the other side.	The left leg is left forward while bending the left knee to 90° while straightening the right leg to the rear and the right arm to the front and resting the left hand on the hip; 2) standing on the left leg while raising the right foot and bending the right knee to 90° and bringing the right hand to the right hip; and 3) repeating on the other side.	The left leg is left forward while bending the left knee to 90° while straightening the right leg to the rear and the right arm to the front (the left hand rests on the hip); 2) standing on the left leg while raising the right foot and bending the right knee to 90° and bringing the right hand to the right hip and straightening the left arm to the front; and 3) repeating on the other side.
**4) Increase muscular strength of the lower limbs during functional squatting movements**	Lifting an imaginary ball from the floor and reaching it out to the side, maintaining the neutral zone of the lumbar spine.	8 repetitions to each side	Deeper squat and range of motion.	Deeper squat and range of motion.	Deeper squat and range of motion.
**5) Achieve normal range of motion in thoracic region, and hip and ankle joints**	Active toe stand and knee bend, keeping heels on the floor.	8–10 repetitions	Hip circumduction (emphasizing flexion and abduction) is performed, with the help of the hands, maintaining the neutral zone in the lumbar spine. • After 5 repetitions change direction and repeat 5 times	The hamstrings stretch, flex, and point to the ankle eight times, maintaining the neutral zone in the lumbar spine.	Starting position: Side lying with flexed hips and knees, reaching up the upper arm and neutral spine. The thoracic spine is rotated backward without pelvic movement.
			“Cat–cow–downwards facing dog”.	“Cat–cow–downwards facing dog”.	“Cat–cow–downwards facing dog”.

Participants will be instructed to maintain neutral spine posture by using a mild contraction of the trunk muscles to achieve a natural range of motion during each exercise [28,29,54].

#### Interventions: Concomitant care, modifications and adverse effects

PNE plus NMS or NMS alone will be implemented without altering the usual care pathways, including medication use, which will continue for both trial arms. The protocol of this study is conservative, and both interventions (PNE and NMS) are low risk [29,30]. However, if patients in the intervention group report severe adverse events (e.g., fainting or dizziness), mild side effects (e.g., physical or mental), or experience severe worsening of their clinical condition (e.g., presenting in a session with a VAS ranging from 8 to 10), they will discontinue the study and be referred for additional treatment to a physician.

The physiotherapist administering the treatment will monitor any adverse events or worsening of the patient’s clinical condition during each session. If any adverse events are present, the principal investigator will be informed, and a weekly list will be compiled to monitor them. Moreover, patients will be instructed to return to the principal investigator if they experience any back pain or adverse effects after the trial. Participants may choose to stop participating in the intervention or study for any reason. They will be informed about the permission, consent, and assent forms, and they can choose to stop participating in the study at any time without any consequences.

#### Outcomes variables

Data will be self-assessed at three measurement time points: before the intervention, after 8 weeks of intervention, and at 6 months follow-up. A researcher blinded to the group allocation will collect, share, and maintain confidentiality before, during, and after the trial, ensuring the privacy of all personal information about potential and enrolled participants and outcomes. Unblinding and the procedure for revealing a participant’s allocated intervention during the trial will only be permissible in case of adverse events.

#### Initial socio-demographic and clinical characteristics of patients

The patients’ demographics and clinical characteristics will be collected at baseline as follows: age, weight, height, body mass index (BMI), radiographic score, marital status or living with a partner, educational level, employment status, duration of symptoms, VAS, disability, number of daily medications used, time of onset of severe back pain, joint replacements, and the number and name of other diseases.

#### Outcomes measures

The primary outcome will be pain intensity [30]. The secondary outcomes will be disability [23], fear-avoidance beliefs during work and physical activity [32], self-efficacy [32], and fear of movement [30].

### Primary outcome

#### Pain

The VAS is an extensively used psychometric response scale that assesses participants’ pain severity. It requires participants to rate their pain severity along a horizontal line with ten points, with 0 and an image of a smiling face indicate no pain, and 10 and an image of pain and discomfort indicate severe pain. The VAS has demonstrated excellent reliability and validity, with a high internal consistency of ICC = 0.91 [23]. The minimal clinically important difference (MCID) for people with CLBP is 1.5 cm, as reported in a previous study [56].

### Secondary outcomes measurements

#### Disability

The Persian version of the Oswestry questionnaire will be used to examine the degree of disability in participants with CLBP [57]. This questionnaire consists of 10 six-option items that assess an individual’s capacity to perform daily activities. Each item assigns a disability score to the activity, ranging from zero (no pain during the desired function) to five (severe pain prevents performance). The Oswestry Disability Index is the sum of the scores of these ten items, multiplied by two, producing a value between 0 and 100. A zero score indicates that a person can perform daily activities without pain. Scores between 1 and 20 indicate mild or minor disability, 21 to 40 indicate moderate disability, 41 to 60 indicate severe disability, and 61 to 80 indicate a debilitating disability. Scores of 81 and higher indicate that a person is bed-bound or exaggerating symptoms. The validity of the Oswestry questionnaire has been confirmed with a Cronbach’s alpha of 75%, and its reliability has been reported with a correlation coefficient of 0.92 [58]. The lowest MCID in individuals with CLBP is 10 points [59].

#### Fear-avoidance beliefs

The Persian version of the Fear-Avoidance Beliefs Questionnaire (FABQ) [60] will be used to assess patients’ fear-avoidance beliefs during physical activity and work. It consists of 16 items related to physical activity (FABQ-PA) and work (FABQ-W) that affect patients with LBP. For FABQ-W, those scoring 34 or higher out of a possible 42 points are less likely to return to work within four weeks. In contrast, a score of 15 out of 24 points on the FABQ-PA indicates fear-avoidance beliefs regarding the physical activities [60]. The Persian version of the FABQ has demonstrated validity and reliability (ICC = 0.80) for measuring fear-avoidance beliefs in patients with CLBP [60]. A variation of 4 points for FABQ-PA and 7 points for FABQ-W represents the MCID in CLBP [61].

#### Self-efficacy

The Persian version of the Pain Self-Efficacy Questionnaire (PSEQ) [62] will be used to assess self-efficacy. The questionnaire is a valid and reliable measure of pain self-efficacy beliefs (ICC = 0.92) [62]. The PSEQ is a 10-item questionnaire ranging from 0 to 60 that assesses patients’ confidence in their ability to perform a range of activities despite pain. For example, it includes items such as: “I can do most of the household chores (e.g., tidying up, washing dishes), despite the pain”, and “I can gradually increase my activity level, despite the pain. Lower PSEQ scores indicate lower confidence levels. The MCID for PSQE is 9 points [63].

#### Fear of movement

The Tampa Scale of Kinesiophobia (TSK) measures fear of movement or kinesiophobia in patients with LBP [64]. The total score on this scale ranges from 17 to 68, with 68 indicating severe fear of movement, 37 indicating fear of movement, and 17 indicating no fear. It has been translated and validated into Persian, showing good reliability (ICC test-retest = 0.86) [65]. The MCID for individuals with chronic musculoskeletal pain is 4.5 points [66].

#### Adherence

The analysis will follow intention-to-treat (ITT) principles, including all patients based on randomization, regardless of whether they have received the primary interventions [37]. The principal investigator will document adherence to the exercise regimen in a weekly report. Moreover, missing data will be reviewed, and sensitivity analyses will be conducted when necessary. During the implementation period of the main intervention, weekly reminder calls and text messages will be sent to participants to remind them of the meetings [67,68].

#### Data management

This RCT will comply with the principles outlined in the Declaration of Helsinki [69]. Written informed consent will be obtained from all participants, and their personal data and consent forms will be stored in locked units at the outpatient clinic for archival purposes. Each participant will be assigned a unique ID number to maintain anonymity, and all data collected on the computer system will be based only on this ID number. The document files, including the ID number data and participant awareness, will be kept separate from the study information files and the consent forms. Only the principal investigator and team members of the study will have access to the participant ID number list and information sheets.

Clinical data will be collected and documented by trained research assessors in our outpatient clinic. Clinical data will be recorded on case report forms and then entered electronically into a secure computer system. This study adheres to accepted clinical practices to safeguard patients’ rights and benefits [69]. All data collected will be validated against the source documents and All data collected will be validated against the source documents and The data will be kept at health Centre for five years after the data collection. All raw data required to replicate the results of your study. Participants will be informed that they may withdraw their consent at any time during the study and that their medical care or legal rights will not be affected.

The Trial Steering Group and independent Data Monitoring and Ethics Committee will meet to review conduct throughout the trial period, once every two months, or as needed based on any reported adverse event. Safety monitoring will begin at the start of trial recruitment. The committee will provide a report detailing all adverse events and protocol deviations, which will be notified and updated in the clinical trial registry. This will be a small study; therefore, there will be no requirement for an interim analysis or stopping guidelines.

### Statistical analysis

The sample size has been estimated for the primary outcome of pain intensity (VAS), with emphasis on the proportion (%) of patients with improved CLBP on the VAS. The minimal important change in VAS is reported to be 15 mm [70]. Accordingly, we expect a minimal difference of 20% between the intervention groups in the proportion of patients with an improved VAS score (at least 15 mm) [27]. To detect a difference in main effects (i.e., PNE plus NMS vs NMS), the sample size calculation using G*Power software and like previous studies in the field of PNE (30), with an effect size difference of 0.25, an alpha value of 0.05 (two-sided test), and power (1 × b) 0.80, results in a required total sample size of 60 participants (30 patients per group). Assuming a dropout rate of 20%, we will enroll 30 patients per group.

A 2 × 2 variance analysis (treatment group × time) will be conducted using a mixed-model analysis design. The percentage change will be calculated for each variable compared to the baseline. The effect size (ES) using partial eta squared (ηp2) will be calculated to measure the clinical meaningfulness [71]. For ITT analyses, group means will replace missing data during the post-test. There will be no additional analyses (e.g., subgroup and adjusted analyses). All data analyses will be conducted using the SPSS software version 26 (IBM Corp., Armonk, NY, USA) at an alpha level of 0.05.

### Plans to give access to the full protocol, participant-level data and statistical code

All the data required to support the protocol will be supplied upon request. All datasets and statistical code requests will be submitted to the corresponding author for consideration. Access to anonymized data may be granted following a consensus involving the trial statistician and Priment Clinical Trials Unit team. No images or other personal or clinical information about the participants are included in this report or will be included in future reports of the trial results. The participants’ information materials and informed consent forms will be obtained from the corresponding author upon request.

### Patient involvement

Patients will be involved in the study planning so that the time for the call (report pain and update exercise), time of the start of the first session of exercise, time to assessment (at baseline, after 8 weeks, and 6 months), (PNE plus NMS and NMS groups) can be adapted according to their preferences, availability, and comfort. We will encourage patients to self-report their feelings about the volume, intensity, or type of exercise, and inform their physiotherapist accordingly.

### Post-trial care and dissemination policy

The interventions involved in this trial are expected to cause minimal harm. There is no anticipated harm or compensation for trial participation. The results obtained will be disseminated at the national and international levels in the form of scientific articles published in relevant peer-reviewed journals.

## Discussion

In this paper, we present a protocol for an RCT that aims to investigate the effects of PNE plus NMS compared with NMS alone on pain, functional and psychological outcomes in 60 patients with CLBP. The synergy between PNE and NMS may provide comprehensive benefits by simultaneously reducing pain, improving functional capacity, and alleviating psychological distress, thus shedding light on the recovery process in patients with CLBP [22,32].

As a strength, this protocol offers a fresh perspective on the management of CLBP. First, combining PNE with NMS addresses both the physical and psychological impairments of CLBP. The combined approach is expected to improve patient outcomes more effectively than either intervention alone, thereby enriching the therapeutic options for CLBP. Second, the protocol design follows international reporting guidelines (SPIRIT, CONSORT, TIDIER) [33–35,72] and adopts single-blind assessments and ITT analysis to ensure rigorous and high-quality data collection and interpretation. Third, the study considers diverse outcome measures (e.g., pain intensity, functional disability, and psychological factors) in accordance with the biopsychosocial model in CLBP [14]. Taken together, these actions represent useful strategies for overcoming the methodological issues presented in RCTs on NMS [27–29], thereby contributing to the advancement of knowledge on this topic.

However, there are several limitations to this study. The sample size, although statistically adequate, may limit the generalizability of the results to the broader CLBP population. Further studies with larger sample sizes are required to confirm our future findings. Another limitation is the monocentric design, as the study will be conducted within a single outpatient healthcare setting and will consider patients with CLBP (not recurrent LBP) [30] exclusively from Iran, impacting the external validity of the findings. Additionally, the lack of a validated Persian version of the Central Sensitization Inventory [22,32] will hinder exploration of the relationship between pain improvement and central sensitization. Furthermore, we have considered patient-reported outcomes (PROs) as primary and secondary outcomes. Although PROs are commonly used in RCTs, they are prone to limitations, such as response bias, recall bias, and the subjective nature of self-reported data [73]. Finally, excluding individuals above the age of 60 and below 30 years old from the study will restrict the applicability of our findings to these specific age groups. Further research should incorporate a wider range of age groups and investigate long-term outcomes to better understand the effectiveness of the interventions.

In conclusion, our RCT can contribute to advancing the management of CLBP and optimizing patient care, thus providing new insights to the rehabilitation field.

### Trial status

The trial is currently underway. This trial is at protocol version 5, dated 17 March 2023. Recruitment of participants commenced on 10 March 2024 and is expected to be completed by February 2025.

## Supporting information

S1 File SPIRIT protocol of the trial. (DOC)

S2 File SPIRIT checklist for Physiotherapy Research International (PRI). (DOCX)

S3 File The TIDieR (Template for Intervention Description and Replication) checklist. (PDF)

## Abbreviations

CLBP: Chronic low back pain
FABQ: Fear-Avoidance Beliefs Questionnaire
NME: Neuromuscular Exercise
ODI: Oswestry Disability Index
PNE: Pain Neuroscience Education
PO: Post-test
PR: Pre-test
PSEQ: Pain Self-Efficacy Questionnaire
RCT: Randomized Control Trial
TSK: Tampa scale for kinesiophobia
VAS: Visual Analogue Scale
