# Supplementary material for: Effects of pain neuroscience education combined with neuromuscular exercises on pain, functional disability and psychological factors in chronic low back pain: A study protocol for a single-blind randomized controlled trial
Source: PLoS One. 2024 Nov 4;19(11):e0309679. doi: 10.1371/journal.pone.0309679 (PMC11534247; doi:10.1371/journal.pone.0309679)
Supplement: S1 File — (DOC) [file pone.0309679.s001.doc]

# **SPIRIT Checklist for *Physiotherapy Research International (PRI)***

|  |  | **Reporting Item** | **Page and Line Number** | **Reason if not applicable** |
| --- | --- | --- | --- | --- |
| **Administrative information** | | | | |
| Title | #1 | Descriptive title identifying the study design, population, interventions, and, if applicable, trial acronym | Page 1 |  |
| Trial registration | #2a | Trial identifier and registry name. If not yet registered, name of intended registry | Page 3, lines 26 & 27  The link for easy access of readers to the trial information: <https://www.irct.ir/trial/69146> |  |
| Trial registration: data set | #2b | All items from the World Health Organization Trial Registration Data Set | Page 3, lines 26 & 27  The link for easy access of readers to the trial information: <https://www.irct.ir/trial/69146> |  |
| Protocol version | #3 | Date and version identifier | Pages 18, lines 361 |  |
| Funding | #4 | Sources and types of financial, material, and other support | N/A | The study has no monetary funding. |
| Roles and responsibilities: contributorship | #5a | Names, affiliations, and roles of protocol contributors | Page 1and page 18&19 lines 377-388 |  |
| Roles and responsibilities: sponsor contact information | #5b | Name and contact information for the trial sponsor | N/A | This is a trial initiated by a research team. The authors had indemnity insurance and legal liability for the study, and the Faculty of Physical Education and Sports Sciences, Kharazmi University, Tehran, Iran is not responsible. |
| Roles and responsibilities: sponsor and funder | #5c | Role of study sponsor and funders, if any, in study design; collection, management, analysis, and interpretation of data; writing of the report; and the decision to submit the report for publication, including whether they will have ultimate authority over any of these activities | N/A | The study has no sponsor. |
| Roles and responsibilities: committees | #5d | Composition, roles, and responsibilities of the coordinating centre, steering committee, endpoint adjudication committee, data management team, and other individuals or groups overseeing the trial, if applicable (see Item 21a for data monitoring committee) | Page 15, lines 299-303 |  |
| **Introduction** |  |  |  |  |
| Background and rationale | #6a | Description of research question and justification for undertaking the trial, including summary of relevant studies (published and unpublished) examining benefits and harms for each intervention | Pages 4-6, lines 31-81 |  |
| Background and rationale: choice of comparators | #6b | Explanation for choice of comparators | Pages 4-6, lines 31-81 |  |
| Objectives | #7 | Specific objectives or hypotheses | Pages 5-6, lines 80-81 |  |
| Trial design | #8 | Description of trial design including type of trial (eg, parallel group, crossover, factorial, single group), allocation ratio, and framework (eg, superiority, equivalence, non-inferiority, exploratory) | Page 6, lines 82-87 |  |
| **Methods: Participants, interventions, and outcomes** | | | | |
| Study setting | #9 | Description of study settings (eg, community clinic, academic hospital) and list of countries where data will be collected. Reference to where list of study sites can be obtained | Page 7, lines 113-116 |  |
| Eligibility criteria | #10 | Inclusion and exclusion criteria for participants. If applicable, eligibility criteria for study centres and individuals who will perform the interventions (eg, surgeons, psychotherapists) | Pages 7 -8, lines 120-131 |  |
| Interventions: description | #11a | Interventions for each group with sufficient detail to allow replication, including how and when they will be administered | Pages 9-10, lines 148-183 |  |
| Interventions: modifications | #11b | Criteria for discontinuing or modifying allocated interventions for a given trial participant (eg, drug dose change in response to harms, participant request, or improving / worsening disease) | Pages 10, lines 184-190 |  |
| Interventions: adherence | #11c | Strategies to improve adherence to intervention protocols, and any procedures for monitoring adherence (eg, drug tablet return; laboratory tests) | Pages 14, lines 274-280 |  |
| Interventions: concomitant care | #11d | Relevant concomitant care and interventions that are permitted or prohibited during the trial | Pages 11, lines 192-194 |  |
| Outcomes | #12 | Primary, secondary, and other outcomes, including the specific measurement variable (eg, systolic blood pressure), analysis metric (eg, change from baseline, final value, time to event), method of aggregation (eg, median, proportion), and time point for each outcome. Explanation of the clinical relevance of chosen efficacy and harm outcomes is strongly recommended | Pages 11-14, lines 195-265 |  |
| Participant timeline | #13 | Time schedule of enrolment, interventions (including any run-ins and washouts), assessments, and visits for participants. A schematic diagram is highly recommended (see Figure) | Page 29 |  |
| Sample size | #14 | Estimated number of participants needed to achieve study objectives and how it was determined, including clinical and statistical assumptions supporting any sample size calculations | Page 15&16, lines 307-316 |  |
| Recruitment | #15 | Strategies for achieving adequate participant enrolment to reach target sample size | Page 35 |  |
| **Methods: Assignment of interventions (for controlled trials)** | | | | |
| Allocation: sequence generation | #16a | Method of generating the allocation sequence (eg, computer-generated random numbers), and list of any factors for stratification. To reduce predictability of a random sequence, details of any planned restriction (eg, blocking) should be provided in a separate document that is unavailable to those who enrol participants or assign interventions | Pages 8, lines 141-147 |  |
| Allocation concealment mechanism | #16b | Mechanism of implementing the allocation sequence (eg, central telephone; sequentially numbered, opaque, sealed envelopes), describing any steps to conceal the sequence until interventions are assigned | Page 8, lines 141-147 |  |
| Allocation: implementation | #16c | Who will generate the allocation sequence, who will enrol participants, and who will assign participants to interventions | Page 8, lines 133-136 |  |
| Blinding (masking) | #17a | Who will be blinded after assignment to interventions (eg, trial participants, care providers, outcome assessors, data analysts), and how | Page 8, lines 136-147 |  |
| Blinding (masking): emergency unblinding | #17b | If blinded, circumstances under which unblinding is permissible, and procedure for revealing a participant’s allocated intervention during the trial | Page 11, lines 199-201 |  |
| **Methods: Data collection, management, and analysis** | | | | |
| Data collection plan | #18a | Plans for assessment and collection of outcome, baseline, and other trial data, including any related processes to promote data quality (eg, duplicate measurements, training of assessors) and a description of study instruments (eg, questionnaires, laboratory tests) along with their reliability and validity, if known. Reference to where data collection forms can be found, if not in the protocol | Page 11, lines 196-201 |  |
| Data collection plan: retention | #18b | Plans to promote participant retention and complete follow-up, including list of any outcome data to be collected for participants who discontinue or deviate from intervention protocols | Page 14, lines 267-280 |  |
| Data management | #19 | Plans for data entry, coding, security, and storage, including any related processes to promote data quality (eg, double data entry; range checks for data values). Reference to where details of data management procedures can be found, if not in the protocol | Page 14&15, lines 282-303 |  |
| Statistics: outcomes | #20a | Statistical methods for analysing primary and secondary outcomes. Reference to where other details of the statistical analysis plan can be found, if not in the protocol | Page 15&16, lines 307-323 |  |
| Statistics: additional analyses | #20b | Methods for any additional analyses (eg, subgroup and adjusted analyses) | Page 15&16, lines 307-323 |  |
| Statistics: analysis population and missing data | #20c | Definition of analysis population relating to protocol non-adherence (eg, as randomized analysis), and any statistical methods to handle missing data (eg, multiple imputation) | Page 16, line 320-321 |  |
| **Methods: Monitoring** | | | | |
| Data monitoring: formal committee | #21a | Composition of data monitoring committee (DMC); summary of its role and reporting structure; statement of whether it is independent from the sponsor and competing interests; and reference to where further details about its charter can be found, if not in the protocol. Alternatively, an explanation of why a DMC is not needed | Page 15, lines 299-303 |  |
| Data monitoring: interim analysis | #21b | Description of any interim analyses and stopping guidelines, including who will have access to these interim results and make the final decision to terminate the trial | Page 15, lines 304 & 305 |  |
| Harms | #22 | Plans for collecting, assessing, reporting, and managing solicited and spontaneously reported adverse events and other unintended effects of trial interventions or trial conduct | Page 14, lines 266 & 272 |  |
| Auditing | #23 | Frequency and procedures for auditing trial conduct, if any, and whether the process will be independent from investigators and the sponsor | Pages 15, lines 299-303 |  |
| **Ethics and dissemination** | | | | |
| Research ethics approval | #24 | Plans for seeking research ethics committee / institutional review board (REC / IRB) approval | Pages 7, lines 102-106 |  |
| Protocol amendments | #25 | Plans for communicating important protocol modifications (eg, changes to eligibility criteria, outcomes, analyses) to relevant parties (eg, investigators, REC / IRBs, trial participants, trial registries, journals, regulators) | Page 15, lines 301 & 303 |  |
| Consent or assent | #26a | Who will obtain informed consent or assent from potential trial participants or authorised surrogates, and how (see Item 32) | Page 7, lines 111 & 119 |  |
| Consent or assent: ancillary studies | #26b | Additional consent provisions for collection and use of participant data and biological specimens in ancillary studies, if applicable | N/A | No additional consent and biological data were collected as part of this trial. |
| Confidentiality | #27 | How personal information about potential and enrolled participants will be collected, shared, and maintained in order to protect confidentiality before, during, and after the trial | Page 14&15, lines 282 -290 |  |
| Declaration of interests | #28 | Financial and other competing interests for principal investigators for the overall trial and each study site | Pages 20, lines 401 |  |
| Data access | #29 | Statement of who will have access to the final trial dataset, and disclosure of contractual agreements that limit such access for investigators | Pages 16, lines 325-328 |  |
| Ancillary and post-trial care | #30 | Provisions, if any, for ancillary and post-trial care, and for compensation to those who suffer harm from trial participation | Pages 16, lines 325-328 |  |
| Dissemination policy: trial results | #31a | Plans for investigators and sponsor to communicate trial results to participants, healthcare professionals, the public, and other relevant groups (eg, via publication, reporting in results databases, or other data sharing arrangements), including any publication restrictions | Pages 17, lines 343-344 |  |
| Dissemination policy: authorship | #31b | Authorship eligibility guidelines and any intended use of professional writers | Pages 19, line 378-388 |  |
| Dissemination policy: reproducible research | #31c | Plans, if any, for granting public access to the full protocol, participant-level dataset, and statistical code | Pages 16, line 325-328 |  |
| **Appendices** | | | | |
| Informed consent materials | #32 | Model consent form and other related documentation given to participants and authorised surrogates | Pages 16, line 329-331 |  |
| Biological specimens | #33 | Plans for collection, laboratory evaluation, and storage of biological specimens for genetic or molecular analysis in the current trial and for future use in ancillary studies, if applicable | N/A | No biological specimens were collected as part of this trial |

It is strongly recommended that this checklist be read in conjunction with the SPIRIT 2013 Explanation & Elaboration for important clarification on the items. Amendments to the protocol should be tracked and dated. The SPIRIT checklist is copyrighted by the SPIRIT Group under the Creative Commons “[Attribution-NonCommercial-NoDerivs 3.0 Unported](http://www.creativecommons.org/licenses/by-nc-nd/3.0/)
