# Supplementary material for: Effects of pain neuroscience education combined with neuromuscular exercises on pain, functional disability and psychological factors in chronic low back pain: A study protocol for a single-blind randomized controlled trial
Source: PLoS One. 2024 Nov 4;19(11):e0309679. doi: 10.1371/journal.pone.0309679 (PMC11534247; doi:10.1371/journal.pone.0309679)
Supplement: S2 File — (DOCX) [file pone.0309679.s002.docx]

**
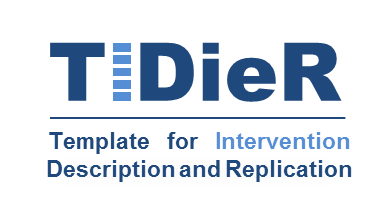
The TIDieR (Template for Intervention Description and Replication) Checklist*:**

Information to include when describing an intervention and the location of the information

| **Item number** | **Item** | **Where located **** | |
| --- | --- | --- | --- |
|  |  | Primary paper  (page or appendix  number) | Other ^†^ (details) |
|  | **BRIEF NAME** | Page 1 | Pain neuroscience training combined with neuromuscular exercises on chronic low back pain. |
| **1.** | Provide the name or a phrase that describes the intervention. |  |  |
|  |  | Page 2, lines 4-8  Page 5, lines 74-78 | The Pain Neuroscience Education (PNE) approach is used to help patients better understand their pain from biological and physiological perspectives. Clinicians also provide it to reduce pain and disability in chronic pain patients. Also, neuromuscular exercises (NMS) are targeted exercises to control impairments presented in CLBP. |
|  | **WHY** |  |  |
| **2.** | Describe any rationale, theory, or goal of the elements essential to the intervention. |  |  |
|  | **WHAT** |  |  |
| **3.** | Materials: Describe any physical or informational materials used in the intervention, including those provided to participants or used in intervention delivery or in training of intervention providers. Provide information on where the materials can be accessed (e.g. online appendix, URL). | Page 9 & 10, lines 154-183 | The goal of PNE is to reconceptualize patients’ negative beliefs about pain. During PNE sessions, information about the nature of pain will also be targeted to reduce kinesiophobia, fear-avoidance beliefs and avoidance behaviour, thereby promoting self-efficacy.  This training includes 8 topics, with oral explanations will be provided with presentation materials. During the training, all the main concepts of the neurophysiology of pain will be explained and discussed.  In this study, NMS will be used for men and women with CLBP. Each training session consists of 3 stages.  1- The warm-up phase for 10 minutes by doing stretching exercises and gentle running in the gym.  2- Performing specific exercises including: exercises to increase spine stability, improve trunk muscle endurance, improve balance, posture control, increase the strength of back muscles and increase the range of motion of the back and pelvis and according to Matalalah protocol for 45 minutes.  3- Cool down the body with exercises and stretching movements for 5 minutes. |
| **4.** | Procedures: Describe each of the procedures, activities, and/or processes used in the intervention, including any enabling or support activities. | Page 31-34 | Pain Neuroscience Education and neuromuscular exercise protocol |
|  | **WHO PROVIDED** |  |  |
| **5.** | For each category of intervention provider (e.g. psychologist, nursing assistant), describe their expertise, background and any specific training given. | Page 8, lines 134-136 | A physiotherapist and sports injury specialist will supervise the interventions. |
|  | **HOW** |  |  |
| **6.** | Describe the modes of delivery (e.g. face-to-face or by some other mechanism, such as internet or telephone) of the intervention and whether it was provided individually or in a group. | Page 7, lines 110-119 | Participants will be informed about this study and how to register through advertisements in physical therapy centres in Tehran (Iran) and social networks. Patients who are interested in participating in the study are asked to contact the contact numbers included in the information and to do their initial registration. Then, on a certain date, the registration of all patients in the health centre and human performance laboratory of Kharazmi University will be completed in person, and qualified people will be included in the study. |
|  |  |  |  |
|  | **WHERE** | Page 7, lines 110-119;  Pages 9, lines 151-153 | Recruitment and registration of patients will be done in a physiotherapy centre and interventions will be done in an exercise room in the same physiotherapy. |
| **7.** | Describe the type(s) of location(s) where the intervention occurred, including any necessary infrastructure or relevant features. |  |  |
|  | **WHEN and HOW MUCH** |  |  |
| **8.** | Describe the number of times the intervention was delivered and over what period of time including the number of sessions, their schedule, and their duration, intensity or dose. | Pages 2, lines 14-20;  Pages 9, lines 149-151 | Interventions will be conducted for 8 weeks and three sessions per week and each session will last for one hour. |
|  | **TAILORING** | N/A |  |
| **9.** | If the intervention was planned to be personalised, titrated or adapted, then describe what, why, when, and how. |  | Not applicable |
|  | **MODIFICATIONS** |  |  |
| **10.^ǂ^** | If the intervention was modified during the course of the study, describe the changes (what, why, when, and how). | Pages 10, lines 184-190 |  |
|  | **HOW WELL** |  |  |
| **11.** | Planned: If intervention adherence or fidelity was assessed, describe how and by whom, and if any strategies were used to maintain or improve fidelity, describe them. | N/A | Not applicable |
| **12.^ǂ^** | Actual: If intervention adherence or fidelity was assessed, describe the extent to which the intervention was delivered as planned. | N/A | Not applicable |

** **Authors** - use N/A if an item is not applicable for the intervention being described. **Reviewers** – use ‘?’ if information about the element is not reported/not sufficiently reported.

† If the information is not provided in the primary paper, give details of where this information is available. This may include locations such as a published protocol or other published papers (provide citation details) or a website (provide the URL).

ǂ If completing the TIDieR checklist for a protocol, these items are not relevant to the protocol and cannot be described until the study is complete.

* We strongly recommend using this checklist in conjunction with the TIDieR guide (see *BMJ* 2014;348:g1687) which contains an explanation and elaboration for each item.

* The focus of TIDieR is on reporting details of the intervention elements (and where relevant, comparison elements) of a study. Other elements and methodological features of studies are covered by other reporting statements and checklists and have not been duplicated as part of the TIDieR checklist. When a **randomised trial** is being reported, the TIDieR checklist should be used in conjunction with the CONSORT statement (see [www.consort-statement.org](http://www.consort-statement.org)) as an extension of **Item 5 of the CONSORT 2010 Statement.** When a **clinical trial** **protocol** is being reported, the TIDieR checklist should be used in conjunction with the SPIRIT statement as an extension of **Item 11 of the SPIRIT 2013 Statement** (see [www.spirit-statement.org](http://www.spirit-statement.org)). For alternate study designs, TIDieR can be used in conjunction with the appropriate checklist for that study design (see [www.equator-network.org](http://www.equator-network.org)).
