# Supplementary material for: Effects of pain neuroscience education combined with neuromuscular exercises on pain, functional disability and psychological factors in chronic low back pain: A study protocol for a single-blind randomized controlled trial
Source: PLoS One. 2024 Nov 4;19(11):e0309679. doi: 10.1371/journal.pone.0309679 (PMC11534247; doi:10.1371/journal.pone.0309679)
Supplement: S3 File — (PDF) [file pone.0309679.s003.pdf]

Effects of pain neuroscience training combined with neuromuscular exercises on pain, functional disability and psychological factors related to chronic low back pain: A study protocol for a randomized controlled trial.

More options

View older revisions Content changed at 2023-12-16, 1402/09/25

Protocol summary

Study aim

Effects of pain neuroscience training combined with neuromuscular exercises on pain, functional disability and psychological factors related to chronic low back pain

Design

In this a double-blind randomized controlled trial, 60 patients (men and women , aged 30 to 60) diagnosed with chronic low back pain will be randomly assigned to one of the following groups: (1) Pain Neuroscience Education plus Neuromuscular Exercises (30 people) and (2) the neuromuscular exercises (30people).

Settings and conduct

All participants will sign a written informed consent before enrolment. Participants will be informed about this study and how to register through advertisements in physical therapy centers in Tehran and social networks.

Participants/Inclusion and exclusion criteria

The patients with CLBP : primary complaints of low back pain. The exclusion criteria are: having any specific pathology in the spine such as disc herniation

Intervention groups

group 1:Pain Neuroscience Education (PNE) plus neuromuscular exercises (NMS) PNE + NMS group patients will receive neuromuscular exercises three times a week along with neuroscience education. Also in this study, NSM will be used for men and women with CLBP. The protocol of NMS used follows the evidence in CLBP field. group 2:neuromuscular exercises (NMS) NMS group patients will only receive neuromuscular exercises without any other intervention three times a week. In this study, NMS will be used for men and women with CLBP. Each training session consists of three stages.

Main outcome variables

-Pain -Disability- Fear-avoidance beliefs-Self-efficacy-Fear of movement

General information

Reason for update

Changing the sample size

Acronym

IRCT registration information

IRCT registration number: **IRCT20190427043384N2**

Registration date: **2023-03-17, 1401/12/26**

Registration timing: **prospective**

Last update: **2023-12-16, 1402/09/25**

Update count: **2**

Registration date

2023-03-17, 1401/12/26

Recruitment status

recruiting

Funding source

Expected recruitment start date

2024-03-10, 1402/12/20

Expected recruitment end date

2025-02-28, 1403/12/10

Actual recruitment start date

empty

Actual recruitment end date

empty

Trial completion date

empty

Scientific title

Effects of pain neuroscience training combined with neuromuscular exercises on pain, functional disability and psychological factors related to chronic low back pain: A study protocol for a randomized controlled trial.

Public title

Effects of pain neuroscience training combined with neuromuscular exercises on chronic low back pain

Purpose

Treatment

Inclusion/Exclusion criteria

Inclusion criteria:

-Persian-native speaker (Male and Female) - Primary complaints of low back pain (more than three months, usually between the lower ribs and the creased part of the buttocks without nonspecific pathoanatomical cause) diagnosed by an experienced physiotherapist and completion of the consent form.

Exclusion criteria:

-previous spinal surgery and neurological signs specific spinal pathology (tumor, infection, fracture, inflammatory disease)

Age

From **30 years** old to **60 years** old

Gender

Both

Phase

N/A

Groups that have been masked

- Participant
- Outcome assessor

Sample size

Target sample size: **60**

Randomization (investigator's opinion)

Randomized

Randomization description

a researcher will generate the allocation sequence with a block size of two using an online random number generator (Random.org). Patients will be randomly assigned into one of two treatment groups in the ratio of 1:1 as follows: PNE plus NMS group (n = 30) or control group (n = 30). Randomization will be drawing a number from 1 to 60, prepared in advance and placed in sealed opaque envelopes in a box. Participants will be told which intervention they were randomized to at the end of the study, after eight weeks.

Blinding (investigator's opinion)

Double blinded

Blinding description

Assessor and biostatistician will be blinded to group allocation. The investigators responsible for data analysis will use a coded dataset to ensure blinding. Patients will not be blinded to exercise study but will not know which treatment group they will be assigned to.

Placebo

Not used

Assignment

Parallel

Other design features

Secondary Ids

empty

Ethics committees

1

Ethics committee

Name of ethics committee

Sport Sciences Research Institute

Approval date

2022-11-21, 1401/08/30

Ethics committee reference number

IR.SSRC.REC.1401.081

Health conditions studied

1

Description of health condition studied

Chronic low back pain

ICD-10 code

M54.5

ICD-10 code description

Low back pain M54.5

Primary outcomes

1

Description

Pain Intensity

Timepoint

Data will be assessed at the three measurement time points from the participants:• Before intervention;• After 8 weeks of intervention• At 6-month follow-up

Method of measurement

In this study, pain will be measured using Visual Analogue Scale (VAS) scale. The VAS, a widely used psychometric response scale tool, measures participants' pain severity. Participants will be asked to indicate the severity of their pain along the VAS, which is a horizontal line with 10 points along it. The beginning shows no pain (marked with a zero and an image of a smiling face), and its endpoint (marked with a 10 and a face in pain and discomfort) represents very severe pain. Its reliability and validity have been excellent, and its internal consistency has been well demonstrated.

Secondary outcomes

1

Description

Disability

Timepoint

Data will be assessed at the three measurement time points from the participants:• Before intervention;• After 8 weeks of intervention• At 6-month follow-up

Method of measurement

In the present study, The Persian version of the Oswestry questionnaire will be used to assess the degree of disability in participants with Chronic low back pain (CLBP) . This questionnaire includes 10 six-option items. These 10 items examine the performance of the individuals in their daily activities. Each item ranks the degree of disability in performance from zero (desired function with no feeling of pain) up to five (disability in performing the activity due to severe pain). The Oswestry disability index is equal to sum of the scores of these 10 items multiplied by 2, the value of which ranges from 0 to 100. A zero score indicates the person can perform daily activities with no pain. A 0-20 score indicates mild or minimal disability, 21-40 indicates moderate disability, 41-60 indicates severe disability, and 61-80 indicates crippling disability. Scores 81 and higher indicate the person is bed-bound or exaggerating symptoms. The validity of Oswestry questionnaire has been confirmed based on Cronbach alpha of 75%, and its reliability has been reported with a correlation coefficient of 0.92 using the test-retest method. The least clinically significant difference (MCD) in people with CLBP is 0.28.

2

Description

Fear-avoidance beliefs

Timepoint

Data will be assessed at the three measurement time points from the participants:• Before intervention;• After 8 weeks of intervention• At 6-month follow-up

Method of measurement

The Persian version of the Fear-Avoidance Beliefs Questionnaire (FABQ) will be used to assess the patients' fear-avoidance beliefs during physical activity and work. It includes 16 items related to physical activity (FABQ-PA) and work (FABQ-W) affecting the patient’s LBP. As for FABQ-W, those with a score >34 or higher (out of a possible 42 points) have shown to be less likely to return to work by four weeks, whereas, in FABQ-PA, those with a score of 15 (out of a possible 24 points) have revealed Fear-Avoidance beliefs related to physical activities. The Persian version of the FABQ has been reported to be a valid and reliable (ICC= 0.80) measure of Fear-Avoidance beliefs in patients with CLBP. The least clinically significant difference (MCD) in people with CLBP is 5.95.

3

Description

Self-efficacy

Timepoint

Data will be assessed at the three measurement time points from the participants:• Before intervention;• After 8 weeks of intervention• At 6-month follow-up

Method of measurement

The Persian version of the Pain Self-Efficacy Questionnaire (PSEQ) will be used to assess self-efficacy. The questionnaire has been found to be a valid and reliable (ICC= 0.92) measure of pain self-efficacy beliefs). The PSEQ is a 10-item questionnaire ranging from 0 to 60 to assess patients’ confidence about their ability to perform a range of activities despite pain. For example: “I can do most of the household chores (e.g., tidying up, washing dishes), despite the pain” and “I can gradually increase my activity level, despite the pain. Lower scores for the PSEQ indicate lower levels of confidence. The least clinically significant difference (MCD) in people with CLBP is 5.5.

4

Description

Fear of movement

Timepoint

Data will be assessed at the three measurement time points from the participants:• Before intervention;• After 8 weeks of intervention• At 6-month follow-up

Method of measurement

The Tampa Scale of Kinesiophobia (TSK) measures “fear of movement” or “kinesiophobia” in the patient. The total score on this scale is between, 17 to 68. For example, a score of 68 showed severe fear of movement, 37 indicates there is fear of movement and where 17 means no fear. It is translated and validated into Persian and has been reported (ICC test-retest = 0.86) (Cronbach’s Alpha was 0.796 in 17 items). The least clinically significant difference (MCD) in people with CLBP is 0.18).

Intervention groups

1

Description

Intervention group: A neuroscience and pain training protocol along with a neuromuscular exercise protocol will be performed three times a week for eight weeks. The neuroscience training protocol includes the following: - Basic explanation of the structure of the brain and nerves, peripheral nervous system and central nervous system. Thresholds of action - Explaining the process of restraining and promoting the spine - Explaining the types of sensations and the transfer process - Explaining the basic concept of neuroplasticity. The neuromuscular training protocol includes the following: - Modified crawl-up exercise - Bird dog exercise - Side bridge exercise - Single stretch exercise Leg - shoulder bridge exercise - Tai Chi warrior exercise

Category

Treatment - Other

2

Description

Control group: they will perform a protocol of neuromuscular exercises three times a week for eight weeks and will not participate in any other treatment plan. The neuromuscular training protocol includes the following: - Modified crawl-up exercise - Bird dog exercise - Side bridge exercise - Single stretch exercise Leg - shoulder bridge exercise - Tai Chi warrior exercise

Category

Treatment - Other
